# Construction Notes

**Published:** 1893-02-18

**Authors:** 


### CONSTRUCTION NOTES.

In commemoration of the forthcoming jubilee of the foundation of the Sheffield Public Hospital and Dispensary, it is proposed to celebrate the event by reconstructing the old fabric. With this in view, the hospital authorities have purchased land for the erection of a new building to contain 170 beds. The excavations for one block have already commenced, Mr. Webster's contract being accepted. The funds in hand, about £15,000, will be almost sufficient for the completion of this block.

